# Sequential Chemoradiotherapy Compared to Radiotherapy in Endometrial Carcinoma

**DOI:** 10.31557/APJCP.2020.21.5.1327

**Published:** 2020-05

**Authors:** Omima Elemam, Seham Abdelkhalek, Doaa Abdelmoety, Engy Aboelnaga, Reem Baraka, Ahmed Zeeineldin

**Affiliations:** 1 *Oncology Center, King Abdullah Medical City, Makkah, Saudi Arabia. *; 2 *Oncology Center, Mansoura University, Mansoura, Egypt. *; 3 *Department of Radiotherapy, Mansoura University, Mansoura, Egypt. *; 4 *Research Center, King Abdullah Medical City, Makkah, Saudi Arabia. *; 5 *Department of Biology, Colorado State University, Fort Collins, USA. *; 6 *NCI, Faculty of Medicine, Cairo University, Cairo, Egypt. *

**Keywords:** Endometrial cancer, adjuvant therapy, sequential chemoradiotherapy

## Abstract

**Background:**

The role of combined modality in the adjuvant treatment of Endometrial Cancer has not been established. This study aims to assess the benefits of Sequential Chemoradiotherapy (SCRT) compared to Radiotherapy (RT) alone in the treatment of patients with Endometrial Cancer.

**Methods:**

Retrospective analysis of patients with Endometrial Cancer stage I to stage III C at King Abdullah Medical city, Makkah. Each group of patients was assigned to receive External pelvic RT, brachytherapy or both. While a second group received SCRT consisting of six cycles of Carboplatin (AUC 5) and Paclitaxel 175 mg/m^2^ followed by radiotherapy.

**Results:**

Fifty-six women were treated of which 26 received SCRT and 30 received RT. The two groups had a median age of 58 years old ranging from 34 – 84 years old with no other statistically significant difference. Patients who received SCRT had poorer prognostic tumor characteris-tics. Median follow-up was 29.6 months (95% CI: 19.6-39.5 months). All deaths (n=5) were exclusively in the RT group. The 2 and 4-year OS rates were 100% and 100% in SCRT group versus 87.3% and 64.9% in RT group (hazard ratio [HR] 0.018 [95% CI: 0-24.4; p= 0.038); The 2- and 4-year DFS were 100% and 100% in SCRT group versus 78.1% and 43.9% in RT group (HR 0.102 [95% CI: 0.103-0.805; p= 0.008).

**Conclusion:**

Adjuvant chemotherapy given before radiotherapy for Endometrial Cancer may lessen the effect of high-risk features on the DFS and OS. Randomized clinical trials are needed to determine the benefits of early Systemic Therapy.

## Introduction

Endometrial Carcinoma is the most common gynecologic malignancy in developed coun-tries and the second most common in developing countries (Siegel et al., 2019). Moreover, it is the fourth common malignancies in Saudi Arabia based on a cancer registry published in 2017. The adjuvant treatment of Endometrial Cancer depends on the risk of relapse which is defined by the cancer stage and the prognostic factors. Low-risk Endometrial Cancer includes women with stage IA grade 1 Endometrial Cancer of endometrioid histology. while high-risk endometrial cancer includes women with stage III or higher Endometrial Cancer, regardless of histology or grade. A Serous Carcinoma, Clear Cell Carcinoma, and Carcinosarcoma are considered at high risk, regardless of the stage. An Intermediate risk includes all others (Colombo et al., 2016). The use of Pelvic external beam radiotherapy has been for many years the standard treatment for high-risk Endometrial Cancer. Patients treated with pelvic radiotherapy showed a delay in pelvic recurrence rate, but with a lack of evidence on survival improvement. On the other hand, chemotherapy was shown to delay distant metastases; However, when compared to external beam radiotherapy, no differences in survival were found (Maggi et al., 2006; Susumu et al., 2008; Greven et al., 2006). PORTEC-3 trial revealed that chemoradiotherapy significantly improved 5-year failure-free survival for patients with high-risk endometrial cancer compared with radiotherapy alone with no significant difference in overall survival (De Boer et al., 2018). Therefore, we aimed to assess the benefit of combined modality Sequential Chemoradiotherapy (SCRT) com-pared to Radiotherapy (RT) in the adjuvant treatment of endometrial Carcinoma in Saudi patients and evaluate their survival outcomes. 

## Materials and Methods

*Study design and participants*

We retrospectively identified patients, with Endometrial Cancer stage I to III C, a historical cohort of endometrial cancer treated at King Abdullah Medical City in Makkah, KSA between July 2011 and July 2018. Patients who did not receive adjuvant therapy or acquired other malignancies within the past 5 years were excluded from the study. The data collected were patients’ characteristics, pathological data, and outcome information attained from the hospital medical records. Moreover, this research protocol was approved by the Institutional Review Board Committee. Since the study performed is retrospective, we obtained a waiver of informed consent from IRB.

The surgery performed was in the form of total abdominal hysterectomy with bilateral salpingo-oophorectomy with or without pelvic, paraaortic lymphadenectomy. For serous, clearcell carcinoma, and carcinosarcoma full surgical staging (with omentectomy, peritoneal biopsies, and lymph node sampling) was done. Patients were treated with either chemoradiotherapy or radiotherapy based on prognostic risk factors. In other words, low-risk patients, defined in the introduction, received radiotherapy only and high-risk patients received chemoradiotherapy.

Patients in the chemoradiotherapy group received six cycles of intravenous carboplatin AUC5 and paclitaxel 175 mg/m^2^ at 21-day intervals. For the second group of patients, they received Chemotherapy followed by RT External pelvic RT (45Gy/5ws/25Fxs), brachytherapy (1200 cGy/25Fxs), or both. The primary end-point was disease-free survival (DFS) and the secondary endpoint was overall survival (OS). DFS was defined as the time between curative surgery and recurrence or death. While OS was defined as the time from diagnosis until death. 

The follow-up regimen included clinical/Pelvic examination every 3/6 months for 2 years then every 6 months or annually. In addition, the CA-125 test was done during each follow-up visit if initially high.

*Statistical analysis*

Statistical analysis was performed using SPSS^® ^version 22 (IBM^©^ Corp., Armonk, NY, USA). Quantitative data were expressed as median and range. Qualitative data were expressed as frequency and percentage. Chi-square test or Fisher’s exact test was used to compare the characteristics between the two groups. The Kaplan-Meier method was used to analyze the disease-free survival and overall survival. The log-rank test was used to compare the two survival curves. Multivariate analysis was done using Cox-proportional hazard regression model. A p value of less than 0.05 was considered statistically significant

## Results

Between July 2011 and July 2018, 56 women were diagnosed with Endometrial Cancer stage I to IIIC and treated (26 received SCRT and 30 received RT). Overall, the median age of patients was 58 years (range, 34 -84 years). Most of the patients had an Eastern Cooperative Oncology Group Performance Status (ECOG PS) of 0-1. There was no statistically significant difference between the RT vs. SCRT groups regarding patients’ characteristics (Table 1) 

Patients who received SCRT had poor prognostic tumor characteristics. They had more advanced stages, higher-grade tumors, deeper myometrial invasion, more non-endometroid histology (p<0.05 for all) (Table 2). 

Patients received either external pelvic and brachytherapy (21%) or only brachytherapy (8%) or external pelvic radiotherapy in the majority of cases (48%). There was good adherence to the proposed adjuvant treatment, with only two patients did not complete the six cycles of chemotherapy due to poor tolerance. All the patients completed the radiotherapy as planned . Febrile neutropenia (≥Grade 3) adverse events occurred in 4 out of 26 patients (15%) who received chemoradiotherapy. Neuropathy (≥grade 2) was significantly more often after chemoradiothera-py than after radiotherapy (eight [30%] patients vs none]. Table 3 summarizes the adverse events and the outcome. 

**Figure 1:**
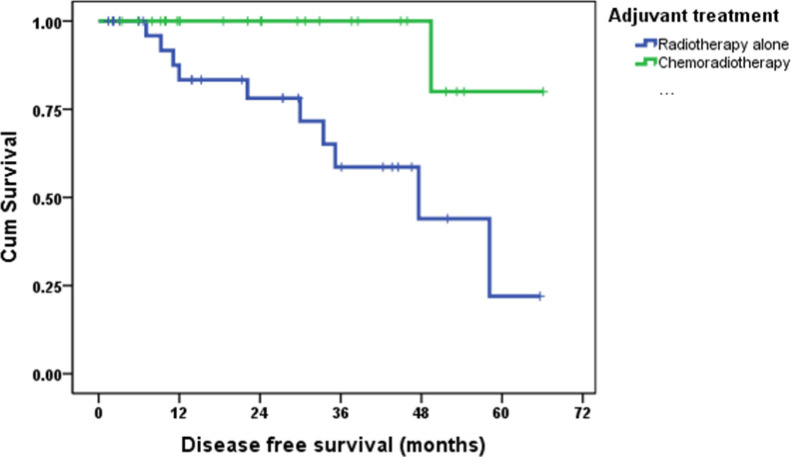
Kaplan-Meier Survival Curve of Disease-Free Survival According

**Figure 2:**
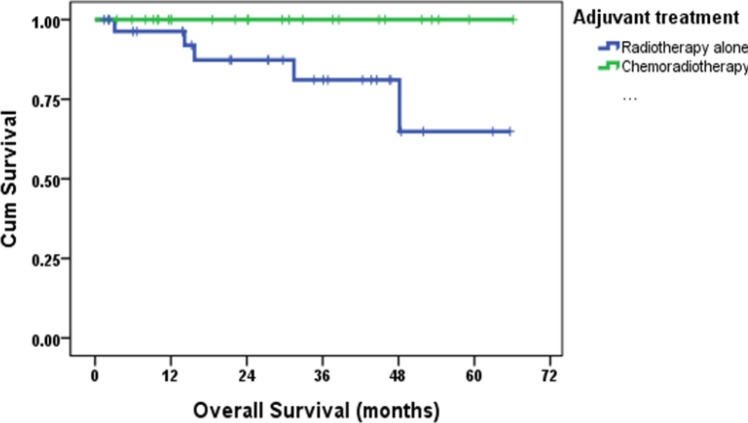
Kaplan-Meier Survival Curve of Overall Survival According to the Treatment between Two Groups

Median follow-up was 29.6 months (95% CI: 19.6-39.5 months). All deaths (n=5) were due to endometrial cancer and exclusively in the RT group. The 2 and 4year OS rates were 100% and 100% in SCRT group versus 87.3% and 64.9% in RT group (hazard ratio [HR]0.018 [95% CI: 0-24.4; p= 0.038); The 2- and 4-year DFS were 100% and 100% in SCRT group versus 78.1% and 43.9% in RT group (HR 0.102 [95% CI: 0.103-0.805; p= 0.008).(Figures 1 and 2)

**Table 1:** Baseline Characteristics of Patients in SCRT and RT Groups

Variable		Total	RT	SCRT	*P-value*
		n=56	n = 30	n = 26	
Age, Median (min-max)		58 (34-86)	58 (34-86)	57.5 (35-75)	-
Median Age	≤58	29 (51.8)	14 (46.7)	15 (57.7)	0.41
	>58	27 (48.2)	16 (53.3)	11 (42.3)	
Elderly	< 65 years	41 (73.2)	21 (70)	20 (76.9)	0.763
	≥ 65 years	15 (26.8)	9 (30)	6 (23.1)	
BMI, Median (min-max)		34.0 (20.5-65.4)	33.8 (20.5-65.4)	34.6 (22.2-54.2)	
BMI (overweight)	Normal	7 (12.5)	3 (10)	4 (15.4)	0.831
	Overweight	9 (16.1)	5 (16.7)	4 (15.4)	
BMI (obese)	Non-obese	16 (28.6)	8 (26.7)	8 (30.8)	0.774
	Obese	40 (71.4)	22 (73.3)	18 (69.2)	
CA125		13.4 (5-184)	12.1 (6-47)	18.42 (5-184)	
Median (min-max)					
Tumor size		5.25 (0.8-14)	4.5 (0.8-10)	6.5 (2-14)	
Median, (min-max)					
PS	1	48 (85.7)	25 (83.3)	23 (88.5)	0.09
	2	4 (7.1)	1 (3.3)	3 (11.5)	
	3	4 (7.1)	4 (13.3)	0 (0)	
PS1	PS 1	48 (85.7)	25 (83.3)	23 (88.5)	0.712
	PS >1	8 (14.3)	5 (16.7)	3 (11.5)	

Data presented as numbers (percentages).

**Table 2:** Baseline Tumor Characteristics of Patients in SCRT and RT Groups

Variable		Total	RT	SCRT	*P value*
		n=56	n = 30	n = 26	
FIGO stage	Stage IA	16 (28.6)	10 (33.3)	6 (23.1)	0.02
	Stage IB	18 (32.1)	12 (40)	6 (23.1)	
	Stage II	10 (17.9)	7 (23.3)	3 (11.5)	
	Stage IIIA	4 (7.1)	0 (0)	4 (15.4)	
	Stage IIIB	4 (7.1)	1 (3.3)	3 (11.5)	
	Stage IIIC	4 (7.1)	0 (0)	4 (15.4)	
Grade	Grade I	13 (23.2)	11 (36.7)	2 (7.7)	0.002*
	Grade II	18 (32.1)	12 (40)	6 (23.1)	
	Grade III	25 (44.6)	7 (23.3)	18 (69.2)	
Grade (1 vs. >1)	Grade 1	13 (23.2)	11 (36.7)	2 (7.7)	0.013
	Grade II-III	43 (76.8)	19 (63.3)	24 (92.3)	
Grade (I-II vs. III)	Grade I-II	31 (55.4)	23 (76.7)	8 (30.8)	0.001*
	Grade III	25 (44.6)	7 (23.3)	18 (69.2)	
Myometrial invasion	≤50%	21 (37.5)	12 (40)	9 (34.6)	0.678
	>50%	35 (62.5)	18 (60)	17 (65.4)	
Histology	Endometriod	40 (71.4)	27 (90)	13 (50)	0.007
	Serous	3 (5.4)	0 (0)	3 (11.5)	
	clear cell	1 (1.8)	1 (3.3)	0 (0)	
	carcinosarcoma	11 (19.6)	2 (6.7)	9 (34.6)	
	Mixed	1 (1.8)	0 (0)	1 (3.8)	
Histology (Endometriod vs. non-endoemtroid)	Endometriod	40 (71.4)	27 (90)	13 (50)	0.001*
	Non_endometroid	16 (28.6)	3 (10)	13 (50)	
Lymphovascular involvement	Negative	29 (51.8)	18 (60)	11 (42.3)	0.193
	Positive	17 (30.4)	6 (20)	11 (42.3)	
	Unknown	10 (17.9)	6 (20)	4 (15.4)	

Data presented as numbers (percentages). *P-value is statistically significant.

**Table 3:** Treatments Administered to Patients in SCRT and RT Groups

Variables		Total	RT	SCRT	*P*-value
		n=56	n = 30	n = 26	
Type of surgery	TAH BSO	39 (69.6)	26 (86.7)	13 (50)	0.004*
	TAH, BSO and Omentectomy	17 (30.4)	4 (13.3)	13 (50)	
Lymph node dissection	Pelvic LN dissection	21 (37.5)	12 (40)	9 (34.6)	0.678
Number of lymph node resected, Median (min-max)		12 (3-23)	12 (9-23)	11 (3-22)	
Radiotherapy Type	EBRT	27 (48.2)	14 (46.7)	13 (50)	0.095
	Brachtherapy	8 (14.3)	7 (23.3)	1 (3.8)	
	Pelvic and Brachytherapy	21 (37.5)	9 (30)	12 (46.2)	
Second line chemotherapy		4 (7.1)	3 (10)	1 (0)	

Data presented as numbers (percentages). *P*-value is statistically significant

**Table 4:** Adverse Events, Metastases and Outcome of Patients in SCRT and RT Groups

Variable		Total	RT	CRT	*P-*value
		n=56	n = 30	n = 26	
Febrile Neutropenia		4 (7.1)	0	4(15.3)	
Neuropathy		8 (14.2)	0(0)	8 (30.7)	
Radiation Proctitis		1 (1.7)	0	1 (3.8)	
Recurrence (local or distant)		11 (19.6)	10 (33.3)	1 (3.8)	0.007*
Locoregional recurrence	Pelvic cavity only	2 (3.5)	2 (6.6)	0 (0)	
	Pelvic LN+ paraaortic	3 (0)	2 (6.6)	1 (3.8)	
	Total	5 (8.9)	4 (13.3)	1 (3.8)	
Distant metastasis	Peritoneal seeding only	1 (1.7)	1 (3.3)	0	
	Lung only	2 (3.5)	2 (6.6)	0	
	Multiple	6 (10.7)	6 (6.6)	0	
	Total	9 (16.07)	9 (30)	0	
Combined local and distant recurrences		2 (3.5)	2 (6.6)	0	
Status of last visit	Alive free of disease	45 (80.3)	20 (66.6)	25 (96.1)	
	Alive with disease	6 (10.7)	5 (16.6)	1 (3.8)	
	Dead	5 (8.9)	5 (16.6)	0 (0)	
OS status	Alive	51 (91.1)	25 (83.3)	26 (100)	0.055
	Dead	5 (8.9)	5 (16.7)	0 (0)	

Data presented as numbers (percentages).*P*-value is statistically significant.

## Discussion

Our results showed that the Adjuvant Sequential Chemoradiotherapy for high-risk Endometrial Cancer (EC) seems beneficial without added toxicity. For years, standard adjuvant treatment for patients with high-risk endometrial cancer was the pelvic external beam radiotherapy. However, results from a recent randomized trial showed that chemotherapy might improve survival by reducing the risk of metastatic disease (De Boer, 2018).

Prospective studies on high-risk early Endometrial Cancer compared observation to radiation therapy, radiation therapy to vaginal brachytherapy, and even radiation to vaginal brachytherapy and chemotherapy. Some studies stated a progression-free survival difference; However, the general evidence is that there is no difference in the overall survival (Miller et al., 2012; Creutzberg et al., 2000; Keys et al., 2004; Nout et al., 2010).

There are several other trials for the high risk and advanced disease that compared chemotherapy to radiation therapy. These trials concluded that there was no difference in overall survival except for GOG 122. The GOG trial showed a difference in favoring five-year progression-free survival, and overall survival in the chemotherapy arm compared to the radiation treatment arm. The NSGO/EORTC MaNGO trial looked at radiation versus radiation followed by sequential chemotherapy that also had a five-year progression-free survival benefit but did not translate to overall survival (Maggi et al., 2006; Susumu et al., 2008; Hogberg et al., 2010; Randall et al.,1995).

In our study, only one recurrence occurred in the patients who received chemoradiotherapy while 4 patients experienced locoregional recurrence and 9 patients experienced a distant recurrence in the radiotherapy group. Furthermore, patients in the SCRT group experienced very low rates of recurrence and the 2- and 4-year OS rates were both at 100%. 

The results, from a large randomized clinical trial [PORTEC-3] that involved 103 centers, revealed the improvement of 5-year failure-free survival in chemoradiotherapy arm in comparison to radiotherapy alone (De Boer et al., 2018). PORTEC3 included women with high-risk EC which involved endometrioid adenocarcinoma, FIGO IB with G3, IA with G3 and lymphatic invasion; endometrioid adenocarcinoma, FIGO II or III; serous or clear-cell EC FIGO I – III) (De Boer et al., 2018). The patients were randomized to either receive pelvic radiotherapy with platinum followed by four cycles of carboplatin and paclitaxel versus radiotherapy alone. There were about 40% in each group who did not have a lymph node dissection performed. While 93% of patients were able to have the first two cycles of cisplatin, fewer patients were able to complete the additional four cycles of carboplatin and paclitaxel. Overall, there was no difference in over-all survival or failure-free survival. Moreover, Distant recurrence was 22% in the chemotherapy arm and 28% in the radiation arm; however, the difference in percentages was not statistically significant. A subset analysis for the patients with only stage III disease showed an improvement in failure-free survival, but there was no overall survival improvement for the chemotherapy and radiation arm in this group. Note, Grade 3 and grade 4 toxicities were significantly observed in the chemo and radiation group. Moreover, one of the biggest weaknesses in PORTEC3 is the heterogeneity of the group that they studied, which may limit the interpretation of the results (De Boer et al., 2018). 

GOG/NRG 258 which was another randomized phase III trial first presented in the ASCO meeting 2017 (Matei et al., 2019). In this study, cisplatin with tumor volume-directed radiation was used instead of just pelvic radiation followed by carboplatin and paclitaxel versus carboplatin and paclitaxel alone. The eligibility criteria were a little bit different than the PORTEC study. In other words, the criteria did not only include surgical stage III and IV disease, but also stage I and II disease was allowed if the patient had high-risk histology. The chemoradiotherapy arm is very similar to the PORTEC study, with the exception of tumor volume-directed radiation. The other arm, around 75% of patients had stage IIIC disease. A higher number of patients in the chemotherapy only group were able to complete chemotherapy compared with the chemoradiotherapy arm. Similar to the PORTEC study, there was no difference in recurrence-free survival and in the overall survival. both vaginal, pelvic, and paraaortic node recurrences were increased in the chemotherapy group. On the other hand, distant recurrences were increased in the radiation group (Matei et al., 2019). 

Another study was the GOG-249 with its initial results were presented in ASTRO 2017 (Randall et al., 2019). The study included 601 patients with EC FIGO stages I and II high-intermediate risks endometrioid EC, serous EC, or clear-cell EC. They received either percuta-neous pelvic radiation (44 Gy/25 fractions or 54 Gy/28 fractions) or vaginal brachytherapy followed by 3 cycles of carboplatin and paclitaxel. In both groups, the 3-year overall survival was (91 vs. 88%). Moreover, the number of vaginal recurrences and distant metastases were (18 vs. 18%) after 5 years. Pelvic and paraaortic recurrences were more common in the combined brachytherapy/chemotherapy group (4% vs. 9%), as was toxicity (≥ grade 3 events in 62% vs. 11%) (Randall et al., 2019). 

These three studies did not give us a conclusive recommendation on the use of adjuvant radio- and chemotherapy. The completion rate of chemotherapy was lower after radiation, raising the question of whether looking at different sequencing of therapy will be important. In our study, we treated the high-risk patients with six cycles of chemotherapy followed by radiotherapy with only two patients not completing the six cycles due to poor tolerance.

For high-risk Endometrial Cancer, especially stage III and IV, the distant metastasis rate, which is a very important endpoint, was lower with chemotherapy. Therefore, systemic therapy needs to be intensified; Potential options are to add more chemotherapy agents either more cycles, a multidrug regimen, immunotherapy, or other systemic options. In other words, to follow through with improving outcomes solely through addressing distant metastases. Moreover, with the recent approval of pembrolizumab from microsatellite unstable tumors, it will be fascinating in future studies to think about looking at pembrolizumab as part of the treatment in these diseases. However, we cannot focus strictly on approaching distant metastasis level, and potentially failing to optimize outcomes by ignoring the importance of regional and local recurrences, especially nodal recurrences. Consequently, the best option is to keep radiation therapy but to continue focus on distant metastases. It was clear that the chemoradiation arm did have an inferior rate of distant metastases. This was the rationale of our study as we treated the patients with a full course of chemotherapy before radiotherapy improving the completion rate of chemotherapy. Therefore, the hypothesis that distant recurrences can actually be impacted if we do chemotherapy first has been supported by all the data.

Moreover, close attention should be focused on the value of the sequence and timing of chemotherapy relative to radiation. There are several options to address this. One could consider a sandwich approach, where half of the chemotherapy delivered first followed by radiation, and then the other half of chemotherapy. However, a clear idea of a randomized controlled trial to support the sandwich approach is not well developed yet. But certainly, the sandwich approach is a technique that physicians use in practice, and evidence from retrospective studies suggest that it may have better progression-free survival than radiation followed by chemotherapy (Secord et al.,2009). Studies should be more focused on the high-risk disease separately with different histologies spread out. In this study, patients with carcinosarcoma were included which is known to be aggressive. Regardless, it did not affect our results that in early stages chemotherapy was effective in preventing replace in our cohort. There are limitations to our study which can be as a result of the risk of bias for retrospective studies. Furthermore, the number of cases comparing two groups who are not equal in tumor characteristics is too small.

In conclusion, the study supports that systemic therapy in the form of chemotherapy performed on patients earlier has great importance in the outcome regarding treating distant metas-tases rates. The high-risk endometrial cancer has at least similar prognosis to low risk endometrial cancer when chemotherapy was used. These results verify the importance of combined radiotherapy and chemotherapy to maximize vaginal, pelvic control, and disease-free survival. Chemoradiotherapy is costlier than radiotherapy alone in terms of toxicity. Looking forward, studies should provide a clear answer to survival benefit. Moreover, more studies on predictive biomarkers in endometrial cancer are needed to identify patients who might benefit from chemotherapy.

## References

[B1] Colombo N, Creutzberg C, Amant F (2016). SMO-ESGO-ESTRO consensus confer-ence on endometrial cancer: diagnosis, treatment and follow-up. Ann Oncol.

[B2] Creutzberg CL, van Putten WL, Koper PC 2000) Surgery and postoperative radiother-apy versus surgery alone for patients with stage-1 endometrial carcinoma: multicentre ran-domised trial PORTEC Study Group Post-Operative Radiation Therapy in Endometrial Car-cinoma. Lancet.

[B3] De Boer SM, Powell ME, Mileshkin L 2018) Adjuvant chemoradiotherapy versus ra-diotherapy alone for women with high-risk endometrial cancer (PORTEC-3): final results of an international, open-label, multicenter, randomized, phase 3 trial. Lancet Oncol.

[B4] Greven K, Winter K, Underhill K 2006) Final analysis of RTOG 9708: adjuvant post-operative irradiation combined with cisplatin/paclitaxel chemotherapy following surgery for patients with high-risk endometrial cancer. Gynecol Oncol.

[B5] Hogberg T, Signorelli M, de Oliveira CF (2010). Sequential adjuvant chemotherapy and radiotherapy in endometrial cancer: results from two randomized studies. Eur J Cancer.

[B6] Keys HM, Roberts JA, Brunetto VL 2004) A phase III trial of surgery with or without adjunctive external pelvic radiation therapy in intermediate risk endometrial adenocarcinoma: A Gynecologic Oncology Group study. Gynecol Oncol.

[B7] Maggi R, Lissoni A, Spina F (2006). Adjuvant chemotherapy vs radiotherapy in high-risk endometrial carcinoma: results of a randomized trial. Br J Cancer.

[B8] Matei D, Filiaci V, Randall M, Mutch D (2019). Adjuvant chemotherapy plus ra-diation for locally advanced endometrial cancer. N Engl J Med.

[B9] Miller DFV, Fleming G, Mannel R (2012). Randomized phase III noninferiority trial of first line chemotherapy for metastatic or recurrent endometrial carcinoma: A Gynecologic Oncology Group study. Gynecol Oncol.

[B10] Nout RA, Smit VT, Putter H (2010). Vaginal brachytherapy versus pelvic external beam radiotherapy for patients with endometrial cancer of high-intermediate risk (PORTEC-2): an open-label, non-inferiority, randomized trial. Lancet.

[B11] Randall M, Filiaci V, McMeekin S (2019). Phase III trial: Adjuvant pelvic radiation therapy versus vaginal brachytherapy plus paclitaxel/ carboplatin in high-intermediate and high-risk early-stage endometrial cancer. J Clin Oncol.

[B12] Randall ME, Spirtos NM, Dvoretsky P (1995). Whole abdominal radiotherapy versus combi-nation chemotherapy with doxorubicin and cisplatin in advanced endometrial carcinoma (phase III) Gynecologic Oncology Group Study. J Natl Cancer Inst Monogr.

[B13] Secord AA, Havrilesky LJ, O’Malley DM (2009). A multicenter evaluation of sequen-tial multimodality therapy and clinical outcome for the treatment of advanced endometrial cancer. Gynecol Oncol.

[B14] Siegel RL, Miller KD, Jemal A (2019). Cancer statistics. CA Cancer J Clin.

[B15] Susumu N, Sagae S, Udagawa Y (2008). Randomized phase III trial of pelvic radiother-apy versus cisplatin-based combined chemotherapy in patients with intermediate- and high-risk endometrialcancer: a Japanese Gynecologic Oncology Group study. Gynecol Oncol.

